# Oocyte quality is decreased in women with minimal or mild endometriosis

**DOI:** 10.1038/srep10779

**Published:** 2015-05-29

**Authors:** Bo Xu, Nan Guo, Xiao-min Zhang, Wei Shi, Xian-hong Tong, Furhan Iqbal, Yu-sheng Liu

**Affiliations:** 1 Center for Reproductive Medicine, Anhui Provincial Hospital Affiliated to Anhui Medical University, Hefei 230001, China; 2 Institute of Pure and Applied Biology. Bahauddin Zakariya University Multan, 60800, Pakistan

## Abstract

Endometriosis, a pathological condition in which the endometrium grows outside the uterus, is one of the most common causes of female infertility; it is diagnosed in 25–40% of infertile women. The mechanism by which endometriosis affects the fertility of females remains largely unknown. We examined the ultrastructure of oocytes from patients with minimal or mild endometriosis and control females undergoing *in vitro* fertilization (IVF) treatment by transmission electron microscopy (TEM) to investigate the physiological significance of oocyte quality for patients with minimal or mild endometriosis. The TEM results revealed that the oocytes from women with minimal or mild endometriosis exhibited abnormal mitochondrial structure and decreased mitochondria mass. Quantitative real time PCR analysis revealed that the mitochondrial DNA copy number was significantly reduced in the oocytes from women with minimal or mild endometriosis compared with those of the control subjects. Our results suggest that decreased oocyte quality because of impaired mitochondrial structure and functions probably an important factor affecting the fertility of endometriosis patients.

Endometriosis is a disorder of the female reproductive system in which the endometrium (uterine lining) grows outside the uterus; it is most commonly occurs on the ovaries and peritoneum, and it causes premenstrual pain and dysmenorrhea^1^,^2^,^3^. The main symptoms of the disease are pelvic pain, dysmenorrhea and dyspareunia^1^. Endometriosis is one of the most common causes of infertility and is diagnosed in 25–40% of infertile women, although the mechanisms by which endometriosis impairs fertility remain largely unknown^2^. The evidence-based therapies for endometriosis include medical and surgical treatments^1^,^2^. Therapeutic laparoscopy and assisted reproductive techniques are the preferred approach for treating infertile patients with advanced endometriosis^2^. In recent years, a substantial number of patients with endometriosis have undergone *in vitro* fertilization (IVF) because the technology of IVF offers a promising alternative to conventional medical or surgical therapies for refractory infertility associated with endometriosis^3^.

The diagnosis of endometriosis in infertile women required the presence of one or more typical bluish or black lesions, and the development stage of endometriosis was determined according to the revised classification of the American Fertility Society (R-AFS), including the implant (ranked according to the diameter and depth of the endometriotic implants) and adhesion scores (ranked according to the density and degree of enclosure). According to the R-AFS scores, the endometriosis divides into four stages: minimal (stage I), mild (stage II), moderate (stage III), and severe (stage IV)^1^,^2^,^3^. The main features of minimal or mild endometriosis are peritoneal or ovarian endometriotic implants and filmy adhesions on the fallopian tubes or ovaries, and minimal or mild endometriosisis more frequently diagnosed than moderate or severe endometriosis^4^. Minimal or mild endometriosis have drawn much more attention from research groups, and the association with IVF outcomes has been extensively investigated^1^,^2^. Several studies have suggested that the presence of minimal or mild endometriosis (stage I/II) is associated with poor fertilization^1^,^2^, whereas the implantation and outcome of pregnancy were implied to be similar in the patients with minimal or mild endometriosis and those having tubal infertility after undergoing IVF treatment^4^,^5^,^6^. Endometriosis, even in a mild stage, because of its poor receptivity, might have a direct negative effect on the oocyte quality, the potential for embryonic development and implantation^7^,^8^.

Dysfunction of a Fallopian tube^9^, subtle impairments of the oocytes and the embryo development potential^10^,^11^, immunological defects^12^ and anatomical dysfunctions of an ovary^13^ have been postulated to explain infertility associated with endometriosis. It is widely accepted that oocyte quality might be a major factor in infertility in these patients^10^,^14^,^15^.

Minimal or mild endometriosis has more frequently been diagnosed than moderate or severe endometriosis in infertile women during *in vitro* fertilization and embryo transfer (IVF-ET) cycles^4^,^7^,^8^. Women with moderate or severe endometriosis typically accept medical and surgical treatments before undergoing IVF-ET treatment, which e causes a concise result to be difficult^7^,^8^. To directly investigate the association between endometriosis and oocyte quality, we enrolled women with minimal or mild endometriosis undergoing IVF-ET treatment in this study. Here, we report the quality of oocytes from patients with minimal or mild endometriosis by using Transmission Electron Microscopy (TEM) and quantitative real time PCR to correlate the oocyte quality of these patients with endometriosis.

## Materials and methods

### Ethical approval

All of the experimental protocols and patient management procedures followed the declaration of Helsinki and were approved by the Ethics Committees on Human Research of Anhui Provincial Hospital, an affiliation of Anhui Medical University, Hefei, China (Notification Number: 2011 Ethics75). The couples consenting to participation in this study signed an informed consent form before being enrolled.

### Subject Information

The enrolled subjects (between 20 and 38 years of age; with tubal disorders and/or a male factor) were IVF patients in the Reproductive Medicine Centre of Anhui Provincial Hospital from February 2011 to October 2012. A total of 41 women who had biopsy-demonstrated endometriosis and had undergone laparoscopic excision of minimal or mild endometriosis [stage I and stage II endometriosis according to the revised classification of the American Fertility Society (R-AFS)] were enrolled in this study (25 patients were enrolled in the TEM analysis, and 16 patients were enrolled in the real time PCR analysis). In this study, the laparoscopy-based diagnosis of endometriosis required the presence of one or more typical bluish or black lesions. The stages of endometriosis were determined according to the R-AFS classification, including the implant and adhesion scores. The implant scores were ranked according to the diameter and depth of the endometriotic implants on the peritoneum or ovaries, whereas the adhesion scores were ranked according to the density and degree of enclosure. Total R-AFS scores (implants and adhesions) from 1 to 5 and 6 to 15 correspond to minimal (stage I) and mild (stage II) endometriosis, respectively. The patients underwent removal of the visible endometriotic implants by excision during laparoscopy. The exclusion criteria were recurrent cysts, polycystic ovary syndrome, endometrioma, uterine adenomyosis and fibroids. Forty homochromous patients without endometriosis detected by diagnostic laparoscopies having tube/male factor based infertility were included as the control group (25 patients were enrolled in the TEM analysis and 15 patients were enrolled in the real time PCR analysis).

### Pituitary down regulation

For all of the patients, a standard long-term pituitary down-regulation protocol was followed. Briefly, all of the patients received GnRH-a (Diphereline; Ipsen Pharma Biotech, Signes, France) down-regulation from the mid-luteal phase of the preceding cycle of gonadotropin (Gn: rFSH, Gonal-F, Merk Serono SA, Geneva, Switzerland) stimulation. The treatment strategy was adapted, according to the ovarian response, followed by detection of the serum follicle stimulating hormone (FSH), the luteinizing hormone (LH), and estradiol (E2) as well as transvaginal ultrasonography, to evaluate whether the pituitary down-regulation was complete. After the pituitary down-regulation was complete, r-FSH injections were initiated. Finally, follicle maturation was induced with 10,000 IU of hCG (LiZhu Pharma, ZhuHai, China) 34–36 hours before oocyte retrieval (when at least 2 follicles of18-mm or more than 3 follicles of 17-mm mean diameter were present). The decision on whether IVF or intra-cytoplasmic sperm injection (ICSI) should be adopted for the patient was determined upon the semen condition on the day of the oocyte retrieval.

### Evaluation of oocytes by Transmission Electron Microscopy (TEM)

A total of fifty mature oocytes (MII) were included in this study. Twenty-five oocytes were collected from 25 patients with minimal or mild endometriosis, and twenty-five oocytes were collected from 25 control women. The oocytes were fixated for four hours following their collection and then processed for the TEM analysis, as previously described^16^. Ultrathin sections (60-80 nm) were cut with a diamond knife, mounted on a copper grid and contrasted with saturated uranyl acetate followed by lead citrate before they were analysed and photographed (JEOL-1230 Transmission Electron Microscope).

### Mitochondrial DNA copy, number determination by quantitative real-time PCR

Nineteen mature (MII) oocytes from sixteen women with minimal to mild endometriosis and eighteen mature (MII) oocytes from fifteen control women were prepared for analysis by quantitative real-time PCR. The cumulus cells of the corona radiata were gradually removed using hand-pulled glass denudation pipettes. The oocytes were washed in NaH_2_PO_4_ (PH2.0) until the zona pellucidae dissolution. The oocytes were frozen in liquid nitrogen for further analysis. The oocyte samples were tested with a SYBR premix ExTaq^TM^ II kit, a quantitative real time PCR reaction mixture composed of 5 μl of SYBR, 0.2 μl of forward-primers, 0.2 μl of reverse –primers, 3 μl of mtDNA template and 1.6 μl of water. The cycling was performed as follows: the initial DNA denaturing step at 95 °C for 10 s followed by 40 cycles, each consisting of denaturation at 95 °C for 5 s and primer annealing at 60 °C for 31 s. The following primer designs were used. The gene segments of ND1 (mtDNA housekeeping genes) was the highly conservative sequence and revealed the total mtDNA in the DNA patterns. The primers, ND1-F 5′- GGCTACATACAATTACGCAAAG -3′and ND1-R 5′- TAGAATGGAGTAGACCGAAAGG -3′, were designed for the assay. After the purification and separation of the PCR product, the internal standard curve was generated from 10-fold dilutions of the standard substance according to the 1 ng PCR products.

### Statistical analysis

In this study, power calculations were performed for the TEM and real time PCR experiments to detect an adequate sample size using the PASS statistical package, version 11. For the TEM experiments, the sample size of approximately 20 patients achieve 100% power to detect the differences between the endometriosis and control groups with a significance level (alpha) of 0.05. For the real time PCR experiments, the sample size of approximately 16 patients achieves 100% power to detect the differences between the endometriosis and control groups with a significance level (alpha) of 0.05. Because our variable was an ordinal level, a statistical analysis of the TEM and real time PCR results was performed with the SPSS, version 13 statistical package. The data were presented as the mean ± sd and compared between the experimental groups with a *t*-test. The rates between the groups were compared using the Chi square test and Fisher’s exact test when appropriate, and P < 0.05 was considered significant.

## Results

### Basic clinical information

In the comparison of the endometriosis and control groups, no significant differences were observed regarding the age, duration of infertility, days of ovarian stimulation, doses of gonadotropins applied and concentration of E2, LH, and progestational (P) on the day of HCG (Table 1).

### Ultrastructure of the oocytes

**Figure 1:**
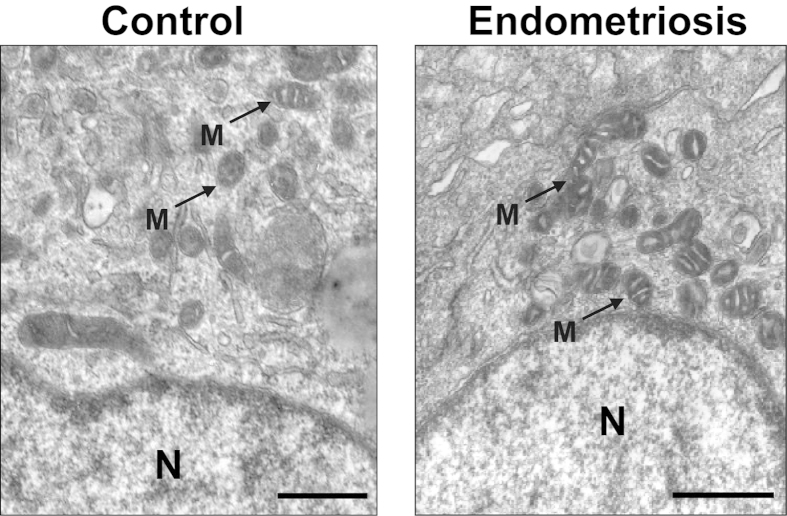
Cumulus cell of the oocytes in the endometriosis and control groups. The cumulus cells from the control group show the same ultrastructural cytoplasmic characteristics of the cumulus cells surrounding the oocytesas in the endometriosis group (**A**,**B**). The tubular cristae of the mitochondria (arrows) are well developed and evenly distributed. N = nuclei; M = mitochondria; Scale bar (**A**,**B**) = 500 nm.

**Figure 2:**
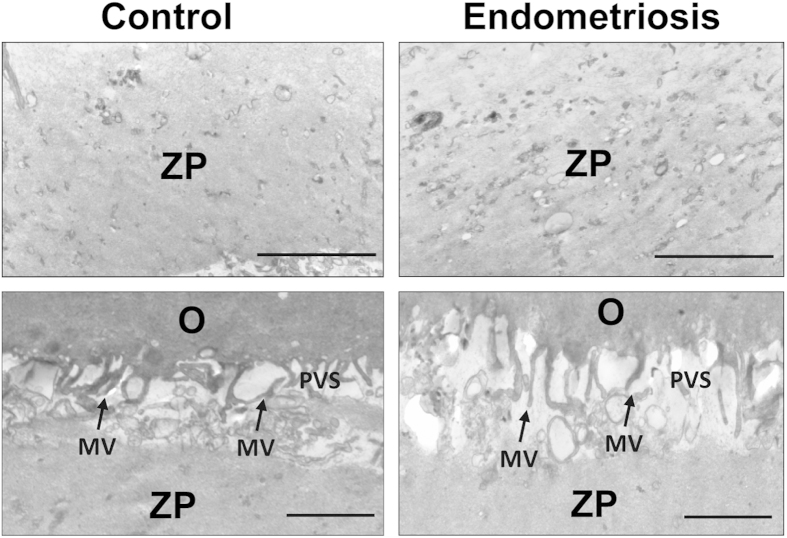
The electron density of the zona pellucida (A,B) and perivitelline space (C,D) in the endometriosis and control groups, respectively. No difference was observed between the endometriosis and control groups in the dense appearance of the inner aspect of the ZP, and some fibres are visible in the zona texture (**A**,**B**). The microvilli (arrows) are numerous and long on the oolemma of both groups (**C**,**D**). MV = microvilli; PVS = perivitelline space; O = oocyte. Scale bar = 500 nm.

The TEM showed that the cumulus cells had abundant organelles and that the cytoplasm of these cells in the endometriosis and control groups had identical bacilli form mitochondria with tubular and/or villiform cristae. The nuclei predominantly contained decentralized chromatin and a voluminous nucleolus (Fig. 1A,B). No difference regarding the density of the filamentous texture of the inner aspect of the zona pellucida (ZP) was observed in the groups (Fig. 2A,B). The oocytes were surrounded by an integrated and regularly structured plasma membrane provided with numerous microvilli stretching into a perivitelline space (PVS) that appeared to be normal in terms of the shape, width and content (Fig. 2C,D). There were no differences in the morphology and electron density of the cortical granule, Golgiapparatus and spindles between the groups (data not shown here).

**Figure 3:**
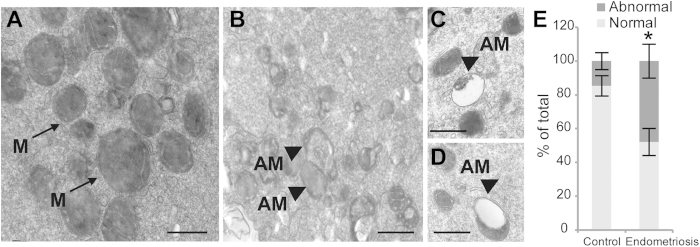
Ultrastructural differences in the mitochondria of normal oocytes and oocytes with endometriosis. Mitochondria with typical tubular cristae are visible in the control cytoplasm (**A**). A large degree of vacuolization (arrows) could be seen in the mitochondria of the endometriosis group (**B**-**D**). The rate of abnormal mitochondria was significantly lower in the control group (**E**). M = Mitochondria; AM = abnormal mitochondria; Scale bar (**A**,**B**,**C**,**D**) = 500 nm.The bars indicate the standard deviation (SD) of the mean. *: compared with those of the control group, the abnormal mitochondria are significantly (P < 0.05) increased in the oocytes from the endometriosis group. Note: Abnormal mitochondria rate = the number of abnormal mitochondria/total number of mitochondria.

**Figure 4:**
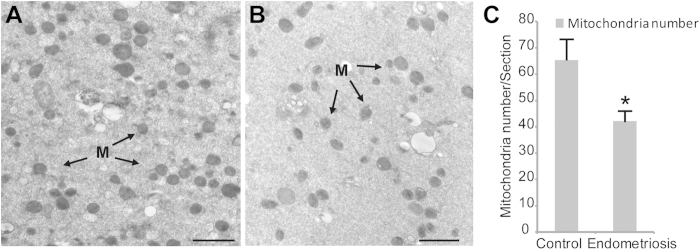
Comparison of the mitochondrial mass in the cytoplasm of the normal oocytes and the oocytes from the endometriosis group. The electron micrograph of the oocytes in the control group revealed abundant mitochondria in the cytoplasm (**A**). However, the number of mitochondria was significantly reduced in the endometriosis group (**B**). There were significant differences between the two groups regarding the mass of the mitochondria in cytoplasm (**C**). The bars indicate the standard deviation (SD) of the mean. *: compared with that of the control group, the number of mitochondria is significantly (P < 0.05) low in the oocytes from the endometriosis group. Note: Mitochondria mass = number of mitochondria/section. For each oocyte, the numbers of mitochondria were counted in at least 3 randomly selected TEM-oocyte sections. To eliminate errors in the mitochondria identification and counting, all of the analyses were performed in a double-blind manner by two or three individuals, and the data were pooled.

In the oocytes of the control group, spherical or elliptical shaped mitochondria were well distributed in the cytoplasm, and arc-like or transverse cristae were irregularly placed on the periphery and parallel to the outer mitochondrial membrane (Fig. 3A). In the cytoplasm of the oocytes from the women with minimal or mild endometriosis, numerous abnormal mitochondria (Fig. 3B–D), which contained small or swollen and blurred vacuoles, were detected. In addition, the percentage of abnormal mitochondria significantly increased in the oocytes of the endometriosis group compared with that of the control group (Fig. 3E). The mass of the mitochondria in the cytoplasm of the oocytes was altered in the endometriosis group (Fig. 4A,B). The relative number of mitochondria within the cytoplasm was significantly decreased in the oocytes from women with minimal or mild endometriosis (Fig. 4C).

### The detection of mitochondria DNA (mtDNA) copies

To ensure the decrease of the mitochondria mass in the oocytes of the endometriosis group, the number of mitochondria was determined by analysing the mtDNA copies. Thus, quantitative real time PCR was performed to analyse the number of mtDNA copies per oocyte from the endometriosis and control group. The control group consisted of 18 mature oocytes (MII) collected from 15 patients with a mean mtDNA copy number of 84,657 ± 39,872 (Table 2). For the 19 mature oocytes of 16 patients from the endometriosis group, the mean mtDNA copy number was 50,781 ± 28,569, which indicated a significantly different mtDNA copy number between the two groups (P < 0.05)(Table 2).

## Discussion

Endometriosis affects a large number of women of reproductive age^17^, and many infertile women with endometriosis select IVF to improve their chances of achieving a pregnancy. Several studies have reported that the rate of fertilization was reduced during IVF/ICSI cycles in patients with minimal or mild endometriosis compared with that of the patients with tubal-factor infertility^7^,^11^,^18^. The mechanism by which endometriosis affects the fertilization rate remains unclear. Infertility in women with endometriosis has been reported to be associated with alterations in normal pelvic anatomy, disturbed hormonal support, ovulation dysfunction and disruption of the development of follicles, oocytes and embryos^19^,^20^. Among the factors associated with infertility in women with endometriosis, the oocyte quality is the most important because it directly reflects the intrinsic developmental potential and is responsible for normal fertilization/embryonic development during IVF. Poor oocyte quality could be the key reason for adverse pregnancy outcomes during IVF/ICSI cycles in women with minimal or mild endometriosis.

Until now, the association between endometriosis and oocyte quality has been detected primarily by clinical data analysis. The IVF-ET cycles in women with endometriosis have been reported to have, in general, a low number of oocytes and decreased fertilization rate^21^,^22^,^23^,^24^. The good embryo rate has been reported to be reduced in the women with endometriosis after stimulated and/or unstimulated cycles of IVF^25^,^26^. Navarro *et al*. reported that the implantation rates of oocytes from donors with endometriosis were reduced in recipients without endometriosis^27^. However, the effect of endometriosis on oocyte morphology has received limited attention. By spindle imaging, Rajani *et al*. suggested that women with endometriosis have oocytes with normal meiotic spindles^28^. Mansour *et al*. documented the morphological characteristics of oocytes by confocal imaging in women with endometriosis and reported abnormal meiotic spindles and chromosomal misalignment^11^. In our study, based on the ultrastructural and quantitative real time PCR analysis of oocytes from patients with or without minimal or mild endometriosis, we have reported that the quality of these oocytes was decreased significantly, possibly because of the alteration of the follicular microenvironment affecting oocyte development and maturation^29^. Mitochondria are double-membrane organelles that play a crucial role in the cell^32^; they are considered to be the powerhouses of the cells and to be involved in diverse signalling pathways and intracellular processes, including regulation of intracellular redox potential, Ca^2^^+^ handling and signalling, mediation of cellular and organismal aging and control of apoptosis^33^,^34^. Mitochondria are hypothesized to be derived exclusively from oocytes, and their activities appear to be essential for oocyte maturation, chromosome segregation and the capacity of a high level of development^35^. Several studies have indicated that mitochondrial abnormalities and/or dysfunction could have an adverse influence on human embryonic developmental and might affect competence for the fertilization of human oocytes^36^,^37^,^38^. In addition, endometriosis lesions, or its secretary products, could result in mitochondria of poorer quality in oocytes, which would affect fertilization and implantation^7^. Therefore, we suggest that minimal or mild endometriosis is specifically linked to the occurrence of impaired mitochondrial structure and reduced mtDNA copy numbers because of disorders of cytoplasmic maturation.

In our study, the mtDNA copy number was decreased significantly in the oocytes of women with minimal or mild endometriosis in comparison to that of the control group (Table 2). Mitochondrial DNA is present in the mitochondrion and in the codes for proteins that are indispensable for cellular energy production^39^.Typically, a normal MII oocyte contains approximately 10^5^ mitochondria in human^40^, and the mtDNA copy numbers could directly represent the mitochondria mass and function^39^,^40^. The low mtDNA content might imply that perturbed oogenesis might be the primaryabnormality responsible for poor oocyte quality. Poor energy production could be linked to insufficient mitochondrial biogenesis and oocyte maturation^39^. Mitochondria with mtDNA that possesses a common deletion are more pervasive in arrested or degenerated oocytes^41^. Additionally, these reports have suggested that mitochondria-related poor oocyte quality is associated with adverse outcomes in IVF/ICSI cycles with minimal or mild endometriosis.

In this study, we examined, by TEM, the oocyte quality in IVF patients with minimal or mild endometriosis; to the best of our knowledge, TEM has not previously been used for investigating the association between oocyte quality and minimal or mild endometriosis. The oocytes from the patients with minimal or mild endometriosis showed increased abnormal mitochondria and reduced mitochondria mass, which suggested that the oocyte quality was decreased in oocytes from women with minimal or mild endometriosis. Some methodological limitations should be noted. In this study, the estimate of the oocyte quality was predominantly based on the ultrastructure analysis. Beyond that, evidence from other aspects was not sufficient. Moreover, only patients with minimal or mild endometriosis were enrolled in this study. For these reasons, these findings could not be generalized to the broader community based on this study alone, and studies using more oocyte quality assessment methods and having patients with different stages of endometriosis are necessary.

## Additional Information

**How to cite this article**: Xu, B. *et al*. Oocyte quality is decreased in women with minimal or mild endometriosis. *Sci. Rep*. **5**, 10779; doi: 10.1038/srep10779 (2015).

## Figures and Tables

**Table 1:** Basic information.

Parameter	Group
	Control Group
**Age (year, mean** ± **std)**	30.31 ± 4.13
**Duration of infertility (year, mean** ± **std)**	4.92 ± 2.15
**bFSH (IU/L, mean** ± **std)**	7.23 ± 2.89
**bLH (IU/L, mean** ± **std)**	4.34 ± 1.81
**bE2 (pg/ml, mean** ± **std)**	45.46 ± 19.42
**bPRL (ng/ml, mean** ± **std)**	14.91±6.27
**Days of ovarian stimulation (mean** ± **std)**	11.93 ± 2.19
**Total of Gn doses (IU/L, mean** ± **std)**	2252.47 ± 828.42
**E2 on HCG day (pg/ml, mean** ± **std)**	2412.24 ± 1379.56
**LH on HCG day (IU/L, mean** ± **std)**	1.14 ± 0.88
**P on HCG day (ng/ml, mean** ± **std)**	1.38 ± 0.12

P < 0.05 was considered statistically significant.

**Table 2:** Mitochondrial DNA copy number for the oocytes from the two groups.

Group	No. of oocytes	Mean	minimum	Maximum
**Control**	19	84,657 ± 39,872	31,100	255,300
**Endometriosis**	18	50,781 ± 28,569^*^	26,900	132,500

Note: The values are the means ± SD. * P < 0.05 vs. the control group.
